# Suicide Made Easy

**Published:** 1915-07-10

**Authors:** 


### SUICIDE MADE EASY.

As suicide is a crime against the State there is a reasonable claim that the pursuit of it, if not actively hindered, shall at least not be facilitated. This claim may be pressed both on the State and on individual citizens, and the neglect of it, though probably technically outside the charge of "accessory before the fact," is not morally very far from this position. If we suppose the organisation of an establishment where lethal chambers, running nooses, and loaded revolvers could be obtained by a visitor on payment of an appropriate fee, it cannot be doubted that self-murder would become more frequent. The proprietor of such an establishment might be in a formal sense outside the powers of the law. But he would hardly enjoy the respect of his neighbours, and the law would promptly be amended to meet his eccentricities. Manifestly such a person would be a promoter of suicide by making the steps to it easy, and he would be rightly condemned as a danger to the community. Even a less active manifestation than this is already forbidden by law. Pharmacists may not distribute active poisons to all and sundry, nor may gunsmiths sell deadly weapons to everyone who proposes a purchase. In short, the law recognises that it has to deal with a certain number of rash and foolish and lackadaisical persons; that some of these under the pressure of the moment will attempt to commit suicide; and that it is the duty both of a civilised community and of every good citizen to hinder rather than to promote the methods by which such act can be accomplished. Whoever fails to discharge this duty becomes directly or indirectly involved in crime. These precautions, it is true, do in some measure interfere with the convenience of many citizens to whom suicide presents no attractions. But this inconvenience has to be suffered lest a worse thing happen to us. The weaker brethren are, indeed, a nuisance, but we cannot altogether ignore them.

The law relating to the sale of poisons is founded on a recognition of the above principles. Yet suicide by poisoning is not uncommon. And at the moment the substance known as veronal is exceedingly popular for this purpose. What is the reason for this selection? First, veronal can be easily obtained. Again, it is known to cause sleep; and the presumption, therefore, is that it will cause an easy or even a pleasant fashion of dying. Last, but by no means least, it is distributed in convenient packages, and in a form in which large doses can be easily and readily swallowed. A neat bottle of tablets involves no question of solution, or of admixture, or of preparation. The position would be very different were the purchaser obliged to face the pharmacist with some discussion of the amount to be purchased, of the cost of this, of the usual dose administered, and of the method of administration. Here would be difficulties not quite easy to overcome. But the present practice removes all these questions and uncertainties, and offers the poison in a form in which its criminal use is encouraged. Such a practice undoubtedly makes suicide a very simple business, and it is impossible to free it from some share of responsibility for the increasing use of the poison. The solution is that no poison should be prepared and dispensed in this subtle and attractive shape except on the written directions of a medical practitioner. If under other conditions persons wish to purchase veronal, let it be in a form in which its misuse is attended with hindrances and difficulties. To sell veronal in convenient tablets, as is the existing custom, is to make suicide easy, and a transaction with such associations ought to be an uncomfortable thing for every person concerned in it. All that the present law demands is that the word "poison," in more or less conspicuous characters, appears on the package. Satisfy this, and apparently it is quite respectable to present the poison in a shape to make self-murder quite simple.

